# "The Hospital" Nursing Mirror

**Published:** 1897-11-27

**Authors:** 


### "The Hospital" Nursing Mirror.

Being the Nursing Section of "The Hospital."

[Contributions for this Section of "The Hospital" should be addressed to the Editor, The Hospital, 28 & 29, Southampton Street, Strand, London, W.C., and should have the word "Nursing" plainly written in left-hand top corner of the envelope.]

### News from the Nursing World.

### THE ROYAL NATIONAL PENSION FUND.

Nurses of the Royal National Pension Fund have sustained a terrible loss through the death of their revered and loved Chairman, Mr. W. H. Burns. It will be difficult for them to express their sympathy adequately with his bereaved family, but no floral tribute will accompany him to his last resting-place from truer mourners than the members of the Royal National Pension Fund for Nurses. The funeral service will be held at St. George's, Hanover Square, on Saturday, at half-past twelve, and wreaths may be sent to 69, Brook Street, W.

### ENGLISH NURSES FROM GREECE.

The two English nurses who served during the Greco-Turkish war, and afterwards volunteered to nurse the thirty typhoid cases sent into hospital at the Piraeus from H.M.S.'s *Rodney*, *Forte*, and *Gibraltar*, are now at home again. Miss Isabel Carter and Miss Catherine Bull have spent the last six months in arduous service, and their late experiences must be full of interest. We wish them a happy holiday now their labours for the time being are ended.

### THE PRINCESS OF WALES AND THE LYNN EPIDEMIC.

The Princess of Wales adds only another example of her warm sympathies with suffering in any shape in writing to the wife of the vicar of Lynn, Mrs. Winter, inquiring after the material comforts and progress of the patients suffering from typhoid, and offering her help in supplying whatever is lacking. Her Royal Highness accompanied her letter by a gift of four dozen night-dresses and flannel bed jackets for the use of the sick. Mrs. Winter, in reply, informed the Princess that the sufferers were well nursed, 15 nurses being employed in the homes of the patients, and others in the hospital. All gifts are deposited in a central store, and distributed as required, The Town Council are ready to engage more nurses if they are wanted, and a committee of ladies superintend the store of invalid necessaries.

### OUR NEEDLEWORK DISTRIBUTION.

Christmas draws on apace, and those kind hands which prepare the useful and beautiful garments for our needlework distribution should be busy with the particular gifts they intend to send us, if our pile of clothing for the destitute in our hospitals is to be in readiness for the festival of goodwill. We look forward to the cheering task of distributing the means of providing creature comforts through the long, cold spring to those whose lives are very barren of things we never lack. The knowledge that our readers sympathise and unite with us in this work is also very helpful and gratifying.

### THE NURSES AT KING'S COLLEGE HOSPITAL.

The nurses at King's College Hospital are well looked after. This year, in honour of the Queen's long reign, each had an extra week's holiday, whilst on Jubilee Day itself one half had the opportunity of seeing the procession, and the other half of enjoying the illuminations. The probationers have two lectures weekly, the one by a member of the medical staff, followed by another on the same subject by the home sister, and at the end of each course an examination is held. The weekly lectures until January 18th, broken, of course, for Christmas holidays, are on anatomy, by Dr. Curnow. Then Dr. Dalton gives ten on physiology; Mr. Carless follows with four on minor surgery; and Dr. Phillips concludes with six on obstetrics. A large number of candidates for training present themselves, but the proportion of suitable ones is small.

### CRIPPLED CHILDREN'S CHRISTMAS.

The Corporation of London, for the fourth time, has granted the use of the Guildhall for the Christmas entertainment of the ragged school children. Alderman Treloar, Ludgate Hill, is interesting himself once more in providing a Christmas hamper for the crippled children of the slums. Last year 1,000 hampers were sent out containing a 2 lb. meat pie, a plum pudding, a cake, a packet of tea, and a parcel of sweets. This year he does not wish to disappoint four out of every five applicants, and boldly asks for funds to send out 5,000. What an army of suffering little ones! It is truly a blessed thing to make one day in the year bright for them, and that day too which the Christ child has hallowed for ever.

### THE CHANCE OF A BARGAIN.

On the occasion of Lord and Lady Aberdeen's visit to Boston, Lady Aberdeen made a speech in which she described the working plan of her new order of district nursing. With her sweet voice and pleading manner she inspired her audience of 3,000—for the most part Canadian-born Bostonians—with such enthusiasm that Dr. Lorrimer said, at the conclusion of her address, "Let's build one of these cottage home hospitals right here and now," and then he sent the collecting-boxes round. Whilst this was being done, he continued—"Then we'll build two or three more outside; and then, by and by, when Lord Aberdeen has got this plan nicely to work in Canada, we'll go over there some day with a plan which we have, and give him a chance to reciprocate." Dr. Lorrimer's bargain is an excellent one for the Canadians; but this putting into words the unwritten law of one good turn deserves another, illustrates in an amusing way the progressive commercialism of the day. A well-bred so-called barbarian would scorn to remind a beneficiary of his obligation upon the bestowal of a gift, no matter how rigorously he would claim his dues later. Civilisation dispenses with ceremony, and clinches a bargain in a business-like way. However, as one good turn certainly does deserve another, the Canadians will doubtless reward Dr. Lorrimer by giving himself and his plan, whatsoever it may be, a cordial welcome whenever he claims it.

### BARROW AND DISTRICT SICK NURSING ASSOCIATION.

Mrs. Wadham has been the moving spirit of this enterprise. She has gained the ear of the townsfolk of Barrow, collected the information and instructed them as to the work of the Queen's nurses amongst the sick poor, and lastly she has gathered about £300 to set the association going. After having done all this she retires now into the background and leaves the committee free to manage its affairs as it thinks best. The meeting called to inaugurate the new association was widely representative and unanimous in its proceedings. Two Queen's nurses are to begin work at once. Lady Evelyn Cavendish has consented to be president, and eight ladies form the committee.

### THE YORKSHIRE CONFERENCE: THE POOR LAW NURSES.

Miss Gibson, matron of Birmingham Workhouse Infirmary, put in a strong plea for workhouse nurses before the Yorkshire Poor-Law Conference lately held at Leeds. She considers that the proportion of trained nurses to inmates is not large enough, and asks guardians to make whatever efforts they could to render the life more attractive to the nurses in their employ. She spoke of the friction caused by lack of adequate supplies of linen and other necessary adjuncts to skilled nursing, and drew a contrast between the full, cheerful life in a nurses' home attached to the training-school and the heavy work, the often uncomfortable accommodation, and the lack of congenial companionship. Mr. Lawson speaking on the same topic advocated putting increased authority into the hands of superintendent nurses. Where a large number of sick were in the infirmary he maintained that it was an advantage for the medical officer and the matron-superintendent to be responsible for the nursing without interference from the guardians.

### THE VICTORIA NURSES' INSTITUTE, CAPE TOWN.

Lady Goodenough opened the Jubilee Commemoration Nurses' Home, Cape Town, on October 22nd. Holland House, Hof Street, has been bought and adapted for the use of the nurses. The speeches made on the occasion traced the origin and carrying out of the movement, which is one of great utility to the town. The unanimous co-operation of the committee formed to carry out the work with the inhabitants of the neighbourhood resulted in the Home being opened free from debt. Business men gave their services, tradesmen supplied things at cost price, and subscriptions flowed in in so cheering a manner that Holland House, which at first seemed too expensive an investment, ultimately became the property of the association at a cost of £4,250. And now work has been begun, the desirability and ways and means of adding district nurses to the staff is already under discussion.

### TWO BACKWARD BOARDS.

At Honiton, where there are thirty patients in the workhouse, they have hitherto utilised the services of one nurse. When the matter of the Local Government Board order was brought up before a meeting of the Guardians, more than one of these referred to the order to appoint an assistant nurse as a mere fad and ridiculous, as there were plenty of paupers who could assist both by day and night. A very candid expression of opinion is reported at a recent meeting of the Royston Board of Guardians. The Clerk, Mr. Simmons, is reported to be of opinion that the Local Government Board "had no power to make them have additional nurses if they did not think it necessary. He thought that with the help they could have (from paupers) one nurse was quite sufficient, and there was no need to run to further expense. The circular (Local Government Board order) was ambiguous, and they were within their rights in interpreting it in the way they pleased." At the Royston Workhouse infectious cases have hitherto been nursed solely by paupers.

### CLONMEL ONCE MORE.

The appointment of Dr. Sheehan's nurse to undertake the instruction of the nuns at Clonmel Workhouse is but the first step in reorganising the whole system until now in force. The workhouse hospital is to become a training school for lay probationers, who will be accommodated in the hospital, and taught by the nuns for three years, and certificated afterwards. Their wages will be first-class rations, part clothing, and about £10 a year in money. Dr. T. J. Crean, the medical officer, is the instigator of the new departure. There is only one point that seems to militate against its utility. Can one nurse train the sisters of mercy so that they are fitted to teach probationers in so short a time? If the training of probationers should begin at once, should it not be with a full staff of trained women? It seems necessary to remind Irish Guardians that it is sometimes wise to hasten slowly.

### WANTED, MALE NURSES.

The Stoneham Board of Guardians want a trained male nurse to attend to the ward of helpless old men. It is out of the question to employ an untrained attendant, and there is a strong feeling rising against women being engaged day after day with these cases. Many other people and institutions would welcome trained male nurses. Many most suitable men desire training, but the difficulty is to find a training school. Which hospital will be the pioneer in this movement? It is bound to come sooner or later, and the honour of inaugurating the advance, which is worth competing for, can only belong to one.

### SHORT ITEMS.

About 200 guests, including the Mayor and Mayoress of Bradford, were present at the seventh annual ball on behalf of the Bradford Children's Hospital. The rooms were gaily decorated, and the comfort and pleasure of the guests appear to have been abundantly provided for.—Mrs. Humphrey-Owen opened a sale of work, which proved successful, in aid of the funds of the Llanidloes District Nursing Association.—There is a strike amongst the pauper wardmaids in the Longford Workhouse. These women had been paid extra rations for their work, and when the Local Government Board put a stop to this, they refused to do the work. Evidently the end of Guardians' troubles is not yet awhile.—Miss Nunn, matron of the Dewsbury and District General Infirmary, was awarded £10 at a recent meeting of the Board of Management. The infirmary has never been so efficiently and economically managed before, and the gift was made in recognition of her splendid service.

### The Late Mr. Walter Hayes Burns,

### CHAIRMAN OF THE PENSION FUND.

We have to record with real sorrow the sudden death of Mr. W. H. Burns, chairman of the Council of the Royal National Pension Fund for Nurses. Mr. Burns has been, for about ten weeks past, under medical care at his house in the country, North Mymms Park, Hatfield. On Friday we learnt in response to our enquiries that he was progressing satisfactorily towards recovery, and his sudden death on Monday evening caused a shock not only to his relatives but to all who had the pleasure of his friendship or acquaintance. So unexpected was his death that when it occurred most of his family were absent from the room, where he was sitting in a chair. Mr. Burns simply placed his head on the back of the chair and passed away. All of us must desire that when our time comes the end may be as peaceful and painless as that of our departed friend.

Mr. Burns was chairman of the Council of the Royal National Pension Fund for Nurses from the first meeting ever held until his death. It is not a little remarkable that this Fund, which has proved such a comfort and stay to British nurses throughout the Empire, should have been started originally through the munificence of an American citizen, the late Mr. Junius S. Morgan, Mr. Burns's father-in-law; that the first meeting ever held in connection with the practical work of the Fund should have been held by the courtesy of the directors in the board-room of the Equitable Insurance Society of the United States in Cheapside; and that Mr. Burns, the first and only chairman the Fund has had, should have also been an American citizen. Blood is thicker than water, and the mere recital of these facts proves to demonstration the intimate ties and relations which cement all sections of the English-speaking race. Such associations must ever make them something more than strangers if something less than brothers. The nurses of the Royal National Pension Fund owed much to the late Mr. Junius S. Morgan, and the debt of gratitude they owe to the late Mr. Burns it would be difficult to express in words. He was a most punctual and regular attendant at the meetings of the Council; to him belongs the credit of the fact that from the date of its inception to the present time there has never been a period, despite the troubles and panics which have affected the money market, in which the investments of the Fund could not have been realised to produce a larger sum than they originally cost; and when to this statement is added the further fact that the average rate of interest earned upon the moneys invested exceeds 4 per cent. per annum, those who know most about such matters will best realise the loving and continuous care expended by Mr. Burns upon the safe keeping and careful investment of the savings of the nurses. Ever ready to help with his purse, Mr. Burns was never backward in using his influence with the wealthier of his friends and acquaintances to secure liberal gifts to the Donation Bonus and Benevolent Funds connected with the Pension Fund. Wise in counsel, conciliatory and resourceful in all his relations with those around him, endowed with a large heart and a wise head, it would be difficult to exaggerate the gap which his death has caused. His colleagues on the Council are deeply grieved and pained at the sudden death of their valued colleague, whom they knew well, and had every cause to trust and admire with all their hearts.

In the City of London, where the business of Mr. Burns's firm—that of J. S. Morgan and Co.—is carried on, Mr. Burns has come to occupy a position second to none. The feeling in regard to his death, which was openly expressed in the City on the day of its occurrence, was that there was probably no one who at the present time was more thoroughly or deservedly trusted, or whose opinion carried greater weight in times of emergency than his. He will be greatly missed there too, for he, as the head of the English house of the great American firm of J. P. Morgan and Co., of New York, wielded great influence in many circles which cannot fail to feel the blow severely for a long time to come. Of recent years, like his late father-in-law, Mr. Burns had taken a great interest in Guy's Hospital, of which he was a governor, and where his services on the Finance Committee were much appreciated. By his influence and example he has been the means of inducing many people to give largely to charity, and for this reason the voluntary charities of the metropolis have good cause to regret his premature death, and to sympathise deeply with the widow and children he has left behind him.

Mr. Junius S. Morgan met with his death on April 3rd, 1890, and in the seven and a-half years since that death occurred, the National Pension Fund, under Mr. Burns's able administration, has increased by leaps and bounds. At that time there was no Junius S. Morgan Benevolent Fund; the Donation Bonus Fund amounted to less than £30,000; whilst the invested funds did not exceed £52,000. At the present time the actual sum invested exceeds £350,000, the Junius S. Morgan Benevolent Fund exceeds £14,000, and the Donation Bonus Fund has increased to upwards of £63,000. It is not surprising that the management which has produced these magnificent results should have won the entire confidence of the great army of nurses and attendants on the sick, and who at the present time pay into the Fund every year upwards of £60,000, a sum which represents the savings which they are able to put by annually against a rainy day. No man could surely desire a better memorial than that which a bare statement of the foregoing facts contributes to the memory of the late Mr. Walter H. Burns.

In him the nurses lose a warm-hearted and sympathising friend, but they have the consolation of knowing that before his death Mr. Burns consented to his eldest son becoming a member of the Council, and that they can therefore look in future with every confidence to Mr. Walter S. M. Burns, who inherits in no small degree the characteristics of his lamented father, and who is equally devoted to the cause of the nurses, and equally determined to do his utmost to strengthen and uphold the Pension Fund.

It would be difficult to exaggerate what the members of the late Mr. Junius S. Morgan's family have done for nurses in this country. Mr. J. Pierpont Morgan who is one of the vice-presidents, has contributed very largely to the Donation Bonus Fund, and also to the Benevolent Fund. He has never failed to show his interest in this movement whenever occasion has offered, and we are confident that anything he or his firm can do to fill the gap caused by Mr. Burns' death will be done *con amore*, and with all the weight of the great influence they so deservedly possess. Mrs. Walter H. Burns, who is one of the patronesses, is also an active member of the committee of the Junius S. Morgan Benevolent Fund, in which she takes a great interest, and nurses owe the badges of which they are so deservedly proud largely to her spontaneous kindness in defraying the initial cost of their production at a time when it was difficult to arrange this matter without trenching upon the funds of the society. The hundreds of nurses who had the privilege of receiving their certificates at the hands of H.R.H. the Princess of Wales, the president, on the last occasion will gratefully remember the kind manner in which Mrs. Burns gave each of them their tickets for Marlborough House at the great gathering at the Queen's Hall, at which she presided. The remembrance of all the many acts of kindness on behalf of British nurses by members of the Morgan family must quicken their natural sympathies, and create a feeling of the most widespread and deep sympathy with Mrs. W. H. Burns and her children in their present period of grievous trial. We hope that a knowledge of this fact may prove a comfort to them in their present sorrow, and that they will be able in some measure to realise the genuine sympathy and the universal desire which exists to do everything or anything in any way to give expression to a feeling which is as spontaneous in its origin as it is honourable to the noble-hearted family who have called it forth.

Originally joining the Council with much diffidence and hesitation, at the urgent request of his father-in-law and the present deputy-chairman, Mr. Burns soon became engrossed in the work, and it is no exaggeration to say that throughout his chairmanship, from the commencement of the Fund to the day of his death, there were few, if any, things with which he was connected in which he took so whole-hearted and genuine an interest. He has fallen asleep at an age when most men are still in active business, and he leaves to his colleagues a great trust and a noble example of good work for others well and faithfully discharged. They will feel that they must accept that trust and follow that example, so that the Royal National Pension Fund for Nurses may ever continue with God's blessing to prosper and extend. In such a case there will soon be not a single nurse or attendant on the sick throughout the British Empire who has not had an opportunity to seek its protection and to secure the comforts and independence which, thanks in a large measure to the late Mr. W. H. Burns, all are guaranteed who have the wisdom to place their savings in the hands of the Council.

### Lectures to Surgical Nurses.

By H. A. LATIMER, M.D. (Dunelm), M.R.C.S. of Eng., L.S.A. of London, Consulting Surgeon, Swansea Hospital; President of the Swansea Medical Society; Lecturer and Examiner of the St. John Ambulance Association, &c.

### XIX.—RELATIVE STATES OF HARDNESS OF BONES AT DIFFERENT PERIODS OF LIFE—FRACTURES: THEIR VARIETIES, MODES OF PRODUCTION, AND SYMPTOMS.

As you would understand, there are a variety of fractures to be met with, and all of them have received appropriate names whereby they may be described. When a bone, for instance, has been broken in one place and no injury has resulted to neighbouring parts, the fracture is spoken of as being a simple one; should some neighbouring part have been injured—say, an artery or a vein be divided, a muscle be torn through, or the lung be pierced—we have to deal with a complicated fracture; if, with the broken bone, a wound extends through the skin, which is also torn, a compound fracture exists; if the bone has been broken into fragments, or bits, the fracture is described as being a comminuted one; and if, whilst the bone is bent it is not completely broken through, a portion of it having given way, whilst another portion has remained intact, it is called a greenstick fracture. You all know how difficult it is to break a stick which has just been cut off a living tree; you can bend it in all directions, and, whilst doing so, some of its outer fibres tear or break, but you cannot succeed in snapping it across as you can do with a stick which is dry and has lost all its elasticity. So it is with a greenstick fracture; the bone is bent and some of its fibres are broken, but it does not snap across as in the other varieties of fractures which I have been speaking of. There is a good reason for this, connected with the age of the person concerned. These greenstick fractures occur only in childhood, because at that period of life bones are more elastic than firm; as people advance in life the reverse obtains, and their bones lose their elasticity and become brittle. In very early life they are little more than appropriately-shaped pieces of cartilage, or layers of membrane; later on earthly salts become deposited in these structures, and they are converted into bone, and the longer we live the more completely these changes from softness to hardness take place. Mr. Frederick Treeves has so admirably described this change of condition, when speaking of the skull at different periods of life, that I give you his own words as an illustration of what I have been saying. He remarks: "The skull of an infant is to that of an old man as a cranium of thin tin would be to a cranium of strong earthenware."

Although blows and falls are generally the causes of the fractures of bones which you will meet with, these fractures may also take place as a result of some disease in the system whereby they have been rendered brittle—and such cases are spoken of as being spontaneous ones. They are rare conditions and hardly concern you as nurses, and I only mention the fact now to explain a term which you may hear used occasionally. And sometimes bones are broken by the sudden and violent contractions of powerful muscles which are attached to them, as in the case of the kneecap bone, which may be snapped across when the strong muscles of the front of the thigh are set in motion. But, ordinarily, fractures result from some violence directed against the bone, either in a direct or an indirect manner; if, for instance, a limb is struck, one or more of the bones in it may be broken opposite to the place where the blow has been received, and then the fracture is said to have been caused by direct violence; or, a person having fallen down, may discover on getting up again that a bone has been broken somewhere distant from the part of his body on which he alighted when he fell: and this fracture is said to have resulted from indirect violence. Such an accident often occurs in this way: a person slips, instinctively puts out his hand, cannot recover his balance, and falls upon the ground upon his hand with his arm outstretched, and one or both of the bones of the forearm, more or less distant from the hand, break from not being able to bear the strain which has been put upon them.

The symptoms resulting from a bone being broken are just what you would expect them to be when you consider what has happened. They need not all be present, but some of them are sure to be met with. We will consider them one by one. In many cases the patient will have felt the bone snap, and on examining the suspected part you will find an irregularity there, and an unnatural movement. The irregularity may be due to a displacement of the bones, which have been broken by the mere force of the blow which has done the injury; or it may result from the divided bone being drawn apart and aside by muscles which are attached to it. This contraction of muscles also leads to another frequently met with symptom—shortening of the limb. More or less pain occurs; and, in the cases of long bones, unless the broken ends have been driven into each other and fixed there (a condition which is known as impaction), a sign can generally be elicited which marks definitely the fracture. This sign is known as crepitus, or, in plain English, grating. It is none other than the rough noise and sensation you would expect to find if two roughened surfaces are rubbed against each other. To obtain it the surgeon holds the suspected limb with his two hands, one placed above the seat of injury and the other below; he then presses the hands together and makes movements from side to side, and if a fracture has taken place, and the broken ends are not fixed, they grate against each other. When the bones have been comminuted, it is needless to say the condition is easily discovered, and when there is a compound fracture it is, as a rule, also far from difficult to diagnose. Compound fractures often result from efforts made to move parts which have been broken, but which have not been properly secured by splints. A man, for instance, falls down and breaks his leg, the bystanders tell him to stand up, and he does so; the unsupported bone now pierces the surrounding soft parts and skin, and what was in the first case a simple fracture is now converted into a serious compound one. When a complication has arisen in any of these fractures it is declared by some additional symptoms to the ones due to the primary condition. The new symptoms are connected with disturbance of function in any part which has been injured, and I will tell you of some such complications when I come to describe special fractures later on in this lecture.

### The Royal British Nurses' Association.

By command of Princess Christian, the president, a special meeting of the General Council of the Royal British Nurses' Association was held at the rooms of the London Medical Society, 11, Chandos Street, Cavendish Square, W., on Friday, the 19th inst., to consider the draft of the new bye-laws. About ninety members of the Council were present.

H.R.H. Princess CHRISTIAN, in taking the chair, said: I think an erroneous view has got abroad that these proposed new bye-laws have been compiled without my knowledge and sanction. I should like to say that I have known of them from the very beginning, and that I entirely approve of them. I will ask Sir Dyce Duckworth to take the chair for me.

Sir DYCE DUCKWORTH then took the chair, and

Mr. E. A. FARDON (hon. medical secretary) proceeded to explain the bye-laws to the meeting. The most important proposals contained in those bye-laws were those which dealt with the constitution of the two governing bodies of the Association, the General Council and the Executive Committee. The General Council by the Charter was the supreme governing body of the Association, and they had endeavoured to make that Council as representative and powerful as possible. As *ex officio* members they proposed that the matrons of hospitals in Great Britain and Ireland to which medical schools were attached, and which maintained the three years' system of training, should have *ex officio* seats on the Council, and that the matrons of other important hospitals with more than 200 beds which maintained the three years' system should also have *ex officio* seats. These two bodies would give about sixty matrons, so that it would be seen that if the bye-laws were adopted they would give the matrons a very preponderating and permanent influence upon the governing body of the Association—a greater influence than would be possessed by any other section of the Council, and a greater influence than they possessed under the present bye-laws. Of the ninety elected members of the Council thirty were to be matrons, thirty sisters and nurses, and thirty medical practitioners. This would give them a Council of from 140 to 150 as against 300, the number of the Council at the present time. In the constitution of the Executive Committee a great change had been made. Under the present bye-laws the committee consisted of *ex-officio* and elected members; under the proposed bye-laws it would consist of 30 members elected from the General Council (10 being matrons, 10 sisters or nurses, and 10 medical men), together with the president and the honorary officers. As the honorary officers had to be elected each year, the Executive Committee, with the exception of the president, would consist wholly of elected members. Under these bye-laws also, sisters and nurses had a representation on the committee, a thing they had never before possessed. (Hear, hear.) The officials had been freely accused of conspiring together to rob the matrons and nurses of all influence in the government of their own association, but he thought he had shown them that such was not their intention. (Applause.) In conclusion, he asked those present to help forward the adoption of those bye-laws. They had been carefully considered by counsel, they were in accordance with the provisions of the Charter, they would free the Association from the tyranny of the old bye-laws from which the Association had suffered so much, and they would enable the Association to carry out those greater purposes for which it was founded. (Loud applause.)

The CHAIRMAN thought the best way of forwarding the business would be to take the bye-laws seriatim and discuss any points which each bye-law might suggest.

Dr. BEDFORD FENWICK said he had a few remarks to make upon the subject of the bye-laws generally. Mr. Fardon had most seriously misled the meeting on several points. He had told them that the matrons of the large schools were put upon the General Council for the first time by the new bye-laws. When the Association was founded the matrons of the large general hospitals and of the leading schools were given seats upon the General Council, and it was by the proceedings in May, 1895, that these seats were taken away. In consequence of that action the matrons had ceased to take the slightest interest in or to work for the Association. While statesmen were trying to draw the Colonies closer to England the nurses were breaking away from the mother country owing to the doings of the Royal British Nurses' Association. (Laughter.) That fact seemed to excite merriment, but to him it seemed a very painful one. As proof of his statement, Dr. Bedford Fenwick said that in 1894 the nurses of Canada, Australia, New Zealand, and the Cape were all anxious to affiliate with the British Nurses' Association: now they had decided to have nothing to do with the Association. Dr. Bedford Fenwick was proceeding to discuss the constitution of the General Council, when

The CHAIRMAN reminded him that he was going into details, which would be discussed later.

Dr. BEDFORD FENWICK: If you rule that I shall not discuss these bye-laws I will not do so any further.

Sir JAMES CRICHTON BROWNE challenged Dr. Bedford Fenwick to show what his strength on the Council was by moving a resolution. (Applause.) As regarded the Colonies, he did not think that Dr. Bedford Fenwick had shown that the action of the Colonial nurses had had anything to do with the affairs of the Association, and he did not think that if, as Dr. Bedford Fenwick stated, independent associations were being formed in the Colonies it was a matter of regret to the Association. Even if Dr. Bedford Fenwick's facts were accurate they did not militate against the subsequent affiliation of the Colonial nursing associations with the Royal British Nurses' Association.

Dr. BEDFORD FENWICK said that he knew Sir James Crichton Browne would never have challenged him to move a resolution had he not known that he (the speaker) stood alone. (Ironical applause, laughter, and cries of "Hear, hear.") That fact also seemed to cause merriment. (A voice: "Quite naturally." Cries of "Bravo, bravo," and renewed laughter.) No doubt it would afford merriment to know that he was the only one on the Council who could not be removed at present, as he was a vice-president. Everybody who had objected to the officials had been eliminated from that Council, and very cleverly eliminated too.

The CHAIRMAN then put each bye-law to the meeting, pausing for remarks after each one. Certain small alterations were suggested.

Mr. JOHN LANGTON proposed that the bye-laws as amended be adopted by the Council.

Dr. BEZLEY THORNE seconded, and the motion was carried, Dr. Bedford Fenwick alone dissenting.

Dr. BEDFORD FENWICK said that he desired to place on record the fact that this complete reconstitution of the Association had been brought before the General Council and had been passed without any discussion at all.

The CHAIRMAN said that every bye-law had been submitted, and had anyone wished to challenge any of the bye-laws, plenty of time had been given him to do so. He must therefore ask Dr. Bedford Fenwick to withdraw that remark. Full opportunity for discussion had been given.

Dr. BEDFORD FENWICK: I did not say there was no opportunity for discussion. I said the bye-laws had not been discussed.

The CHAIRMAN: Then may I ask you to state what you consider is the significance of that fact?

Dr. BEDFORD FENWICK said that the significance was this, that whilst he was discussing the bye-laws the chairman stopped him. He would have liked to have told the Council that these new bye-laws were a reconstitution of the Association, and that in more than one instance they infringed the Charter.

The CHAIRMAN: Why did you not tell us then?

Dr. BEDFORD FENWICK: Because when I was discussing them you asked me not to speak to the bye-laws. ("Oh!")

The CHAIRMAN: I gave every member ample opportunity to speak to each separate bye-law. (Hear, hear.) You were referring when I stopped you specifically to a certain bye-law, and I then said you would have an opportunity later on.

Dr. BEDFORD FENWICK: Precisely.

The CHAIRMAN: And you were given your opportunity later on.

Sir JAMES CRICHTON BROWNE asked if these bye-laws had been submitted to counsel, and if so, whether, in counsel's opinion, they were absolutely in conformity with the spirit of the Charter.

Mr. J. LANGTON said that they were.

The CHAIRMAN intimated that that answered Dr. Bedford Fenwick's remark as to infringing the bye-laws, and the proceedings then terminated.

### Presentations.

MISS TOMKINS, Assistant Matron of Lambeth Infirmary, was presented with a silver-fitted travelling bag and rug, and a purse containing money, on Tuesday, 16th inst. As many nurses as could be spared from the wards were present, and the chaplain, the Rev. G. H. Leale, expressed the universal regret at her retirement. Miss Tomkins has worked conscientiously and successfully for 18 years at the infirmary, and carries with her the affectionate regard and good wishes of all who know her.

NURSE MARY LAIT has been presented with a handsome silver-fitted dressing bag from the surgeons, a silver cream jug and sugar basin from the house surgeon and nurses, a silver butter knife from the patients, and a china tea service from the servants of the Newark Hospital, Notts, as a token of love and regard on the occasion of her resigning her post as head nurse, where she has for eight years been highly esteemed by all. Her genial manner and her good work will be greatly missed.

MISS A. G. MACVICAR, Matron of the Royal Scottish Nursing Institution, Queen Street, Edinburgh, has been presented with a handsome silver tea service and tray, with an illuminated address, from the nurses. Miss Macvicar's bright, genial manner and uniform kindness has won the hearts of the nursing staff, and it was with great regret that they heard of her resignation through ill-health.

MISS LOUISA CAREY, Night Superintendent of the Camberwell Infirmary, has been presented by the former and present members of the medical and nursing staffs with a completely fitted nurse's bag. The gift is more especially acceptable at the present moment in consequence of the vague charges recently alleged against Miss Carey and some of her colleagues by the Guardians, and to which they have not yet been given an opportunity of replying.

### Where to Go.

ROYAL BRITISH NURSES' ASSOCIATION.—The Right Hon. Sir John Lubbock, M.P., has kindly consented to deliver the first sessional lecture of the Royal British Nurses' Association on Wednesday, December 1st. The lecture will be given in the rooms of the Zoological Society, 3, Hanover Square, W., at eight p.m., on the subject, "Ants." A limited number of seats will be available for the public.

NATIONAL ORTHOPÆDIC HOSPITAL.—On December 3rd, at two p.m., the Duchess of Marlborough will open the annual sale of work in support of the Aid Fund, which provides additional comforts to the patient. The sale will be continued on the two following days.

ROYAL FREE HOSPITAL.—Dickens' "Christmas Carol" will be read by Sir Squire Bancroft on December 17th, 1897, at a quarter past eight, at Holborn Town Hall, Gray's Inn Road, in aid of the hospital funds.

WORKHOUSE INFIRMARY NURSING ASSOCIATION. — St. Martin's (small) Town Hall on December 2nd, at three p.m. E. Boulnois, Esq., M.P., in the chair.

### "The Members' Rights Defence Committee."

On November 13th we received the enclosed letter, accompanied by an extract from the *Nursing Record* of that date, headed "Another Protest":—

46, York Street, Portman Square, Nov. 13th, 1897.

Sir,—By the desire of the "Members' Rights Defence Committee," I am forwarding to you the enclosed paragaragraph. As your correspondent, "Nurse Louisa East," in your issue of the 6th of November, made a statement with regard to my committee which is untrue, I must ask you, as a matter of justice, to insert in your next issue the paragraph which I now enclose.—I am, sir, yours faithfully,

Margaret Breay,
Hon. Sec. Members' Rights Defence Committee.

To this letter we replied as follows:—

The Hospital, 28 and 29, Southampton Street, Strand, W.C.

Madam,—We shall be happy to publish this week a letter in reply to that of Nurse Louisa East, if it is delivered here by five p.m. to-morrow (Wednesday). We are of opinion that it should contain the names of the members and officers of the Members' Rights Defence Committee. We cannot publish the paragraph you enclose, which has already been published elsewhere, We have further to call your attention to the editorial notice at the head of the correspondence column, in which the letter of Nurse East appeared in The Hospital for November 8th, 1897.—We are, yours faithfully (Signed) The Editor.

Miss Margaret Breay, Honorary Secretary, Members' Rights Defence Committee, 46, York Street, Portman Square.

On November 20th the following paragraph appeared in the *Nursing Record*:—

"QUITE SO."

To the Editor of the *Nursing Record*.

Madam,—In The Hospital newspaper, on November 6th, there was published a letter from "Nurse Louisa East," upon which you commented on November 13th. The Members' Rights Defence Committee, which met last week, indignantly denied the truth of "Nurse East's" statements by a resolution which they passed, and which you were good enough to publish. I sent that resolution also to The Hospital, my committee expecting that as that journal had published Miss East's statement, it would also publish the committee's denial of it. I have to-day received a letter from The Hospital, in which the Editor says: "We cannot publish the paragraph you enclose." No better illustration of the journalistic methods of the paper could, I suppose, be given, than thus to publish false statements, and then suppress their refutation. But, having refused to insert the official reply made by the body attacked, the Editor informed me that he would publish a letter in reply to Miss East if it were sent to him within twenty-four hours, and suggesting that it should contain information for which he had no right to ask. It will be obvious to your readers that no one member of my committee would be justified, or competent, to make any satisfactory reply to the attack, especially when the official answer of the committee was denied publication.—I am, Madam, yours faithfully,

Margaret Breay,
Hon. Secretary, Members' Rights Defence Committee.
November 17th.

We print the whole of the correspondence, because we think that no better illustration of the methods of the society which bears the above grandiloquent title could be given in a ready form for reference. Thus, when members of Parliament and others unacquainted with its methods are importuned to join hands with this association, it is well they should know the kind of proceedings they will be required to countenance. It is hardly necessary to point out that the paragraph referred to headed "Another Protest" was not sent to The Hospital and to the *Nursing Record* simultaneously from the committee of the Members' Rights Defence Committee, but came to us as a reprint from the *Nursing Record*, where it had already received the publicity which we are accused of denying to it. Although we declined to republish old matter, we expressed our willingness to follow the usual proceeding of publishing a letter in reply to our correspondent, but this offer was not accepted. Further, in respect to the statement made by Miss Breay that the Editor asked a question outside his rights, we must point out that Miss Breay's letter would have been of no importance unless she acted as representing a committee when writing, and we naturally wished to know something of the committee she refers to.

### Everybody's Opinion.

**[Correspondence on all subjects is invited, but we cannot in any way be responsible for the opinions expressed by our correspondents. No communication can be entertained if the name and address of the correspondent is not given, as a guarantee of good faith but not necessarily for publication, or unless one side of the paper only is written on.]**

### HOUSEKEEPING.

A "District Nurse" writes: A "Constant Reader" does not state if she lives in town or country. If in the latter she could live much cheaper, more especially if her cottage had a garden where vegetables and fruit grew. Groceries, &c., should be had in fairly large quantities from some London stores; it would be found much cheaper than dealing at the nearest town. If the nurse has to do her own cooking an oil stove should be used, except, perhaps, once a week, when a good supply could be made for the ensuing days, such as pastry, cake, joints, which could afterwards be made into mince or a cottage pie. The writer is, of course, supposing the nurse a handy cook, but how she is going to do for herself and work twelve hours is a problem. It is certain she could not afford to pay anyone except for cleaning, perhaps, once a week. An eight hours days is considered long enough for a "Queen's," and all district nurses should, if possible, become "Queen's," then they would have a living wage, though not then overpaid.

"M. D." writes: If the enclosed account is any use to your correspondents, "H. B." and "A Constant Reader," they are welcome to it. I do all my own housework and needlework, part of my washing, &c. I work as required, night or day, in my district, sometimes sixteen and more hours a day, and sometimes only a few hours, using the easy time to pick up my needlework, cleaning, reading, visiting, &c. I do not ask my fellow-nurses to copy me, as the life is too hard for most, and I simply wish to show that it can be done, if they wish to do it, as I have done it many years; but then I am over forty, and perhaps they are young. I may add that sometimes an only brother sends me help with my premiums, amounting to about £3 to £5 a year; but this is not regular, so I dare not count upon it. Salary, monthly, £5. Monthly Expenditure: Rent, £1 4s.; premiums, N.P.F., £1 6s.; washing, 4s.; stationery, books, &c., 4s.; coals, 4s. 8d.; wood, matches, &c, 9d.; milk, 2s. 4d.; bread, 2s. 4d.; quaker oats, 10d.; tea, 1s. 6d.; coffee, 1s.; cocoa, 7d.; butter, 2s. 8d.; dress, travelling, cakes, fruit, &c., £1 5s. 4d. Total, £5.

### NURSES IN GREECE.

"The Chaplain of H.M.S. 'Gibraltar'" writes: A letter has appeared in your columns from someone who signs herself "An English Nurse in Athens," in which it is stated that the nurses who came out from England to help during the war were in the habit of neglecting their duty for the sake of pleasure. In another article in which you comment on a letter from a gentleman in Athens, this charge is repeated, and apparently regarded as rendered worthy of credit from certain things which are said to be common report in Athens. Such a proof is at all times a very worthless one, and to anyone who knows what Athens is like, and especially what was its condition during the war, the common report would have no value whatever. The ship in which I was then serving—H.M.S. "Nile"—was at Phalerum during the war and for some time after the armistice. During that time I had the pleasure of the acquaintance of the nurses, and abundant opportunities of inspecting the hospitals and seeing how the nurses worked. As a result of this personal observation, I say without hesitation that any charges of neglect of duty on the part of the nurses are totally unfounded. The work was very heavy and done under many difficulties, in the midst of surroundings wanting in many of the comforts and conveniences of an English hospital. That they stuck so well to their work and gave up so much for the sake of it, is much to their credit, and the nurses and those who sent them out have good reason to be proud of the way they worked. The patients were full of gratitude for their services, one man assuring me that by their care they saved his life. I hope that in fairness to the nurses you will find room for this, and that soon we shall be favoured with a withdrawal of these unfounded charges.

### Victoria Commemoration Club,

29, SOUTHAMPTON STREET, STRAND, W.C.

THE Secretary desires to draw attention to the fact that nurses can become members of the club now and enjoy all its advantages until December, 1898, for the small sum of 25s. On and after January 1st next the subscription will be one guinea and the entrance fee one guinea. Those nurses joining at once gain a clear advantage of 17s. In spite of its being still in its infancy, and the great difficulty in reaching the class specially interested, the club has already a membership of over 300. Full particulars, with sketches, will gladly be sent by the secretary on receipt of a stamped and addressed envelope. Owing to the short time that has elapsed since its foundation, it has been impossible to make the club universally known. Nurses can help very greatly by bringing the club and the present small fee before their friends and colleagues, and are cordially asked to do so. Admission to Mrs. Alting-Mees' lecture on "Klondyke," on December 2nd, at three p.m., at the above club, is—members, 1s.; non-members, 1s. 6d.

### Minor Appointments.

MIDDLESBROUGH WORKHOUSE.—Miss E. Rainford, the new Superintendent Nurse here, was trained at Chorlton Union Hospital, Manchester, and has been afterwards charge nurse at the Ormskirk Union Infirmary, and under the Metropolitan Asylums Board.

FOUNTAIN FEVER HOSPITAL, LOWER TOOTING.—The new Charge Nurse is Miss Emily Ellis, who was trained at Marylebone Infirmary, Notting Hill. Her previous engagements have been at the Temperance Hospital, as sister at the Holland Nursing Ins'itute, and in private work in Italy.

SOLIHULL UNION WORKHOUSE.—Miss Ellen Partner, who was trained at East Dulwich Infirmary, and charge nurse at the Western Fever Hospital, Fulham, has been elected Head Nurse of this workhouse.

WORKHOUSE INFIRMARY, HIGH WYCOMBE.—On October 4th Miss Mary Baker was appointed Nurse of this institution. She was trained at Brownlow Hill Infirmary, and has subsequently been engaged in private nursing.

ROYAL INFIRMARY, WINDSOR.—Miss Beatrice Reuter, who was trained at King's College Hospital and afterwards staff nurse there, has been elected Charge Nurse of the above.

### Appointments.

### MATRONS.

EAST GRINSTEAD ISOLATION HOSPITAL. — Miss Isabel Barber, the newly-elected Superintendent-Nurse here, was trained at the Children's Hospital, Manchester. She has held the following posts successively: Sister at the Clinical Hospital, Manchester, the Bolton Infirmary, the Blackburn Infirmary, and the Monsall Fever Hospital, Manchester. She has also been engaged in temporary work of different kinds.

### Notes and Queries.

*Our Usual Custom.*

(68) Can you correct a misapprehension that may arise from the way Miss Margaret Ann Clarke's appointment is inserted in the "Mirror" of last week? She is now in the sixth year of her nursing career, and her appointment was gained through her experience and other qualifications than those mentioned.—*J. Clark.*

We can only insert appointments from the particulars supplied us, and our space is too valuable to permit additions and corrections later. We should be pleased to receive full particulars in the first instance, and nurses would help us greatly by supplying them at once on entering upon a new appointment.

*Medicines.*

(69) Would you kindly tell me what medicines would be best to get for a cottage hospital to keep in store? There is no dispensary at it. It is a small place of ten beds. For example, cascara, seidlitz powders, &c.—*Nom de Plume.*

Ask the medical officer for a list of the medicines he would like to have in readiness.

*Uniform.*

(70) Would you kindly tell me if a nurse can claim her uniform, cloak and bonnet, &c., when leaving a district after working there two years, as agreed upon, as uniform given has been counted as part of the salary? —*Uniform.*

If the uniform itself is part of the salary it, or its equivalent value, belongs to the nurse. If, however, only the use of the uniform was part of the salary, then it would belong to the committee.

*Surgical Chart.*

(71) Will you kindly tell me where I can procure an up-to-date surgical operation chart, enumerating the instruments, &c., required for performing ordinary major and minor operations?—*Nurse Grey.*

There are no standard charts. Surgery advances in such strides, and every surgeon has his own method, that the new chart of to-day would be obsolete to-morrow. You might find "Surgical Ward Work," by Miles (The Scientific Press, 28 & 29, Southampton Street, Strand), price 3s. 6d., useful for your purpose?

*Dispensing for Ladies.*

(73 I should be much obliged if you could tell me—(1) How long it takes for a lady to qualify as a dispenser? (2) The probable cost of training to one who is not a nurse? (3) Could you give the address of a training school in Cambridge or in Manchester, preferably in Cambridge?—*A. W.*

Write to the Secretary, Apothecaries' Hall, Blackfriars, E.C., or to the Secretary of the Pharmaceutical Society of Great Britain, Bloomsbury Square, W.C., for information on all points. "Burdett's Annual" for 1894 goes fully into the subject. See also "Mirror," April 3rd, 1897.

*County Council Lectureship.*

(74) I am anxious to try for a lectureship for the County Council Would you kindly tell me where to apply for particulars?—*Adelaide G.*

If you are trained write direct to C. C. for papers; if not trained, Miss Lamport, 52, St. John's Wood Road, N.W., prepares candidates.

*L O.S. Examination.*

(75) Could you kindly give me Miss Lamport's address? (2) Is there any means by which I could pass the L.O.S. examination without going through the three months' course? Can you name a training school where the fees do not exceed £6 6s.. as nurses' fees will not allow such high terms as many ask?—*G. M. Miller.*

(1) See answer 74. (2) Certainly not. (3) Write to Midwives' Institute, 12, Buckingham Street, Strand. We believe that the East London Mothers' Home is the cheapest. It costs about £15 15s. The L.O.S. is cheap at the price.

### Wants and Workers.

[THE attention of correspondents is directed to the fact that "Helps in Sickness and to Health" (Scientific Press, 28 & 29, Southampton Street, Strand, London, W.C.) will enable them promptly to find the most suitable accommodation for difficult or special cases.]

CAN you tell me of a home in London, with board and attendance, for a female domestic servant, old, and rather infirm? Payment can be made.—Amy, care of E. A. Wade. 13, Seymour Street, W.

NURSE NEEDHAM, 97, Ashfield Road, Altrincham, Cheshire, is anxious to hear of a convalescent patient requiring a comfortable home in the country.

